# Potassium Fertilization Stimulates Sucrose-to-Starch Conversion and Root Formation in Sweet Potato (*Ipomoea batatas* (L.) Lam.)

**DOI:** 10.3390/ijms22094826

**Published:** 2021-05-01

**Authors:** Yang Gao, Zhonghou Tang, Houqiang Xia, Minfei Sheng, Ming Liu, Shenyuan Pan, Zongyun Li, Jingran Liu

**Affiliations:** 1 Institute of Integrative Plant Biology, School of Life Sciences, Jiangsu Normal University, Xuzhou 221116, China; 2020170806@jsnu.edu.cn (Y.G.); 2020180451@jsnu.edu.cn (H.X.); 2020201410@jsnu.edu.cn (M.S.); pan-shenyuan@jsnu.edu.cn (S.P.); 2 Jiangsu Key Laboratory of Phylogenomics & Comparative Genomics, School of Life Sciences, Jiangsu Normal University, Xuzhou 221116, China; 3 Xuzhou Sweetpotato Research Center, Xuzhou 221131, China; zhonghoutang@sina.com (Z.T.); 20171004@jaas.ac.cn (M.L.)

**Keywords:** sweet potato (*Ipomoea batatas* (L.) Lam.), potassium fertilization, storage root, biomass production, sucrose-to-starch conversion

## Abstract

A field experiment was established to study sweet potato growth, starch dynamic accumulation, key enzymes and gene transcription in the sucrose-to-starch conversion and their relationships under six K_2_O rates using Ningzishu 1 (sensitive to low-K) and Xushu 32 (tolerant to low-K). The results indicated that K application significantly improved the biomass accumulation of plant and storage root, although treatments at high levels of K, i.e., 300–375 kg K_2_O ha^−1^, significantly decreased plant biomass and storage root yield. Compared with the no-K treatment, K application enhanced the biomass accumulation of plant and storage root by 3–47% and 13–45%, respectively, through promoting the biomass accumulation rate. Additionally, K application also enhanced the photosynthetic capacity of sweet potato. In this study, low stomatal conductance and net photosynthetic rate (*Pn*) accompanied with decreased intercellular CO_2_ concentration were observed in the no-K treatment at 35 DAT, indicating that *Pn* was reduced mainly due to stomatal limitation; at 55 DAT, reduced *Pn* in the no-K treatment was caused by non-stomatal factors. Compared with the no-K treatment, the content of sucrose, amylose and amylopectin decreased by 9–34%, 9–23% and 6–19%, respectively, but starch accumulation increased by 11–21% under K supply. The activities of sucrose synthetase (SuSy), adenosine-diphosphate-glucose pyrophosphorylase (AGPase), starch synthase (SSS) and the transcription of *Susy*, *AGP*, *SSS34* and *SSS67* were enhanced by K application and had positive relationships with starch accumulation. Therefore, K application promoted starch accumulation and storage root yield through regulating the activities and genes transcription of SuSy, AGPase and SSS in the sucrose-to-starch conversion.

## 1. Introduction

At present, sweet potato (*Ipomoea batatas* (L.) Lam.), as a typical storage root crop, is the seventh most essential food crop in world, and is mainly used to produce starch and alcohol and to feed animals [1]. The sweet potato storage root (SPSR) yield depends on the number and the fresh weight of SPSR per plant, and is closely related to root growth and development and SPSR differentiation [2]. The differentiation of SPSR begins at 10–20 days after transplanting (DAT). The adventitious roots grew out of the stem base, and differentiate and enlarge into enlarged roots, and the number of SPSR per plant remains basically stable around 35 DAT [3]. For the fresh weight of SPSR per plant, starch is the major form of fixed carbon in SPSR, approximately 50–80% of SPSR’s dry biomass [4,5]. Therefore, the dry matter and starch in SPSR influence the sweet potato yield.

Potassium (K) is one of the necessary nutrients, and the most crucial osmotic factor in crops [6,7]. As is well-known, K can improve crop stress resistance by optimizing leaf photosynthesis, root elongation, regulation of stomatal guard cells, assimilate transport from source to sink, protein synthesis and enzyme activation [6,7,8,9]. Sweet potato is an important storage crop with higher K requirements for optimum production than other commercial crops [10]. In recent years, much attention has been paid to K fertilizer application in sweet potato production. K application increased chlorophyll content and net photosynthetic rate (*Pn*) in sweet potato leaves [11] and enhanced the photosynthetic products distribution in SPSR and the expansion rate of SPSR [12]. However, excessive K application caused high K intake in sweet potato and a decrease of the K utilization rate [13]. Although there are some reports on the effect of K application on SPSR yield, the studies on its dynamic accumulation are not perfect.

Starch, as the most key carbohydrate in tuber and root crops, is biosynthesized by the decomposition of sucrose products [14]. The conversion from sucrose to starch is regulated by a number of key enzymes. In detail, sucrose phosphate synthase (SPS, EC 2.4.1.14) is a critical enzyme for synthesizing sucrose, and regulating the distribution and conversion of photosynthate between sucrose and starch; invertase (EC 3.2.1.26) and sucrose synthase (SuSy, EC 2.4.1.13) are the main enzymes for sucrose breakdown and energy provision in tuber and root crops [15]; ADP-glucose pyrophosphorylase (AGPase, EC 2.7.7.27) catalyzes ADP-glucose synthesis, used for the synthesis of starch polymers; soluble starch synthase (SSS, EC 2.4.1.21) is essential for amylopectin content, used as a starch branching enzyme (SBE, EC 2.4.1.18); lastly, SBE is essential for amylose content [16,17]. These metabolizing enzymes play crucial roles in the conversion from sucrose to starch and their encoding genes are closely related to starch production [5,17]. 

There are different opinions on the influence of K application on starch and sugar in SPSR. Some studies believe that K application increased the content of starch and sucrose in SPSR [12], which is related to higher AGPase and SSS [11]; other studies believe that K application has little effect on starch content in SPSR [18,19], but rather that it increased the SPSR yield. There is also a view that low K was conducive to the accumulation of starch and sucrose [20]. Although many studies have reported on the correlation of starch synthesis with related enzyme activities [11,12,19], whether and how K application at basal enhances starch accumulation by regulating sink activity, from the perspective of the related enzymes activities and genes transcription in the conversion from sucrose to starch in SPSR, is still unclear. Therefore, we investigated the activities of SPS, SuSy, AGPase, SSS and SBE and the transcriptional levels of their encoding genes in sweet potato plants treated with K, and their relationship with starch accumulation and storage root yield. Two other possible enzymes for sucrose decomposition in SPSR, acid invertase (AI) and alkaline invertase (AKI), were also detected for the activity changes.

There were significant differences among varieties or lines of sweet potato with regards to the effect of K on the SPSR. After fertilization, some varieties increased amylose content, soluble sugar content and amylase activity, while some varieties decreased them [20,21,22]. Additionally, the response of K during SPSR development was significantly different between low-K-tolerant varieties and low-K-sensitive varieties [23,24]. For low-K-tolerant cultivars, K deficiency decreased the total root length, total volume, total surface area, mean diameter and root activity slightly, whereas these parameters decreased about 2–3 times for low-K sensitive cultivars, compared with low-K-tolerant cultivars. In this study, two sweet potato cultivars, Ningzishu 1 (sensitive to low-K) and Xushu 32 (tolerant to low-K) [24,25], were used to study the physiological mechanisms of K_2_O rates effecting SPSR yield. The main objective was to 1) study the development and growth of the SPSR and 2) investigate the regulation of K in the enzyme activities and the transcriptional levels of their coding genes in the conversion from sucrose to starch, and the relationship of these enzymes with leaf K content.

## 2. Results

### 2.1. Photosynthetic Parameters in Sweet Potato Leaves under Different K_2_O Rates

With the increase of the K_2_O rate, leaf *Pn*, *gs* and *Ci* all increased firstly and then decreased, and the peak value was for a treatment with 150–225 kg K_2_O ha^−1^ (Table 1). Compared with the no-K treatment, at 35 DAT, the 75 and 150 kg K_2_O ha^−1^ treatments improved *Pn* by 0.4–12%, while excessive K_2_O slightly decreased *Pn*. At 55 DAT, the application of 75–375 kg K_2_O ha^−1^ increased *Pn* by 3–25%. The trends of *gs* and *Ci* were similar to that of *Pn*. The analysis of variance showed that *Pn* and *gs* had significant differences between cultivars and K_2_O rates, and the CV of Ningzishu 1 among the six K_2_O rates was lower than that of Xushu 32.

With the increase of the K_2_O rate, *Ls* in sweet potato leaves decreased or decreased first and then increased (Table 1), and the variation range of Non-*Ls* was significantly higher than that of *Ls*. Compared to the no-K treatment, the *Ls* and Non-*Ls* values in Xushu 32 leaves after the 75–375 kg K_2_O ha^−1^ treatments were decreased by (−19)−2% and (−23)−22%, respectively, and those in Ningzishu 1 were reduced by (–4)–21% and (−31)−38%, respectively. Therefore, with the increase of the K_2_O rate, leaf *Pn* in sweet potato was more affected by non-stomatal limitation factors.

The difference between the *Ls* value and Non-*Ls* value in sweet potato leaves was only found between cultivars and among K_2_O rates (*p* < 0.05, Table 1), and the CV of Ningzishu 1 among the six K_2_O rates was higher than that of Xushu 32. However, the interaction effect between cultivars and K_2_O rates was not stable due to the influence of years.

### 2.2. Dynamic Accumulation and Simulation of Sweet Potato Biomass

**Figure 1:**
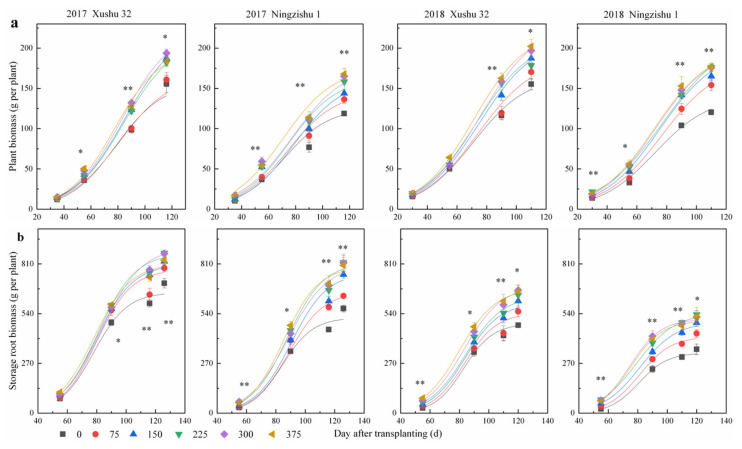
Sweet potato biomass accumulation of plant and storage root under different K_2_O rates in 2017 and 2018. (**a**) Plant biomass accumulation; (**b**) storage root biomass accumulation. Each data point is the mean ± SE of at least three replications. * and ** mean significance among the different K_2_O rates at the *p* < 0.05 and 0.01 probability levels, respectively.

In sweet potato, plant biomass increased following a logistic model by DAT, and significant differences were observed among the six K_2_O rates (Figure 1a). With the increase of the K_2_O rate, the maximum growth rate appears later (*Tm*), and the period of rapid plant biomass increase (*T*) was first prolonged and then slightly shortened, but it was still longer than that of no-K treatment, resulting in a high biomass per plant (Table 2). Compared to no-K treatment, the plant biomass after the 75–375 kg K_2_O ha^−1^ treatments for Xushu 32 and Ningzishu 1 increased by 3–30% and 15–47%, respectively. There were significant differences between cultivars and among K_2_O rates, but their interactions were not observed. In terms of the CV among the six K_2_O rates, the CV of *V_T_* was higher than those of *Tm* and *T*.

Little biomass accumulation in SPSR was observed before 60 DAT; nevertheless, the biomass accumulation in SPSR increased rapidly after 60 DAT, i.e., the SPSR expansion period. There were significant differences among the six K_2_O rates, the biomass after the 75–375 kg K_2_O ha^−1^ treatments in SPSR accumulated with a quicker speed than that of no-K treatment (Table 3), and consequently, the SPSR biomass at harvest was increased with the K_2_O rate increasing, with peak values for the 225–300 kg K_2_O ha^−1^ treatments (Figure 1b). Additionally, the CV of *V_T_* was higher than those of *Tm* and *T*, except that of *T* in Xushu 32.

The dynamic accumulation of plant biomass in sweet potato per DAT was simulated using the Equations (1)–(3), and differences were found in the accumulation of SPSR biomass for the six K_2_O treatments (Table 2). The no-K treatment or 375 kg K_2_O ha^−1^ treatment lasted for the shortest *T* and the lowest *V_T_*. The 150 kg K_2_O ha^−1^ treatment caused a 20–37% higher *V_T_* and a 1.8–3.5 d longer *T* compared to no-K treatment, and the 300 kg K_2_O ha^−1^ treatment caused a 22–40% increased *V_T_* and a 0.9–7.0 d longer *T* compared to no-K treatment.

Differences were also observed among the six K_2_O treatments with regards to SPSR biomass accumulation (Table 3). When all treatments were averaged, the accumulation duration of SPSR biomass was 17.5 d shorter, but faster than that of plant biomass. The 75–375 kg K_2_O ha^−1^ treatments caused a 15–21% higher *V_T_* and a 3.8–5.3 d longer *T* compared to no-K treatment.

### 2.3. Distribution of Sweet Potato Biomass

The accumulation and distribution of sweet potato biomass were remarkedly affected by years, cultivars, and K_2_O rates (Table 4). K application enhanced biomass production and the partition ratio to SPSR. Compared with no-K treatment, the averaged biomass after the 75–300 kg K_2_O ha^−1^ treatments were increased by 7–26% for Xushu 32 and 21–44% for Ningzishu 1, respectively, and the averaged partition ratio to SPSR after the 75–300 kg K_2_O ha^−1^ treatments were increased by 5–12% for Xushu 32 and 7–17% for Ningzishu 1. 

### 2.4. Yield Components

**Figure 2:**
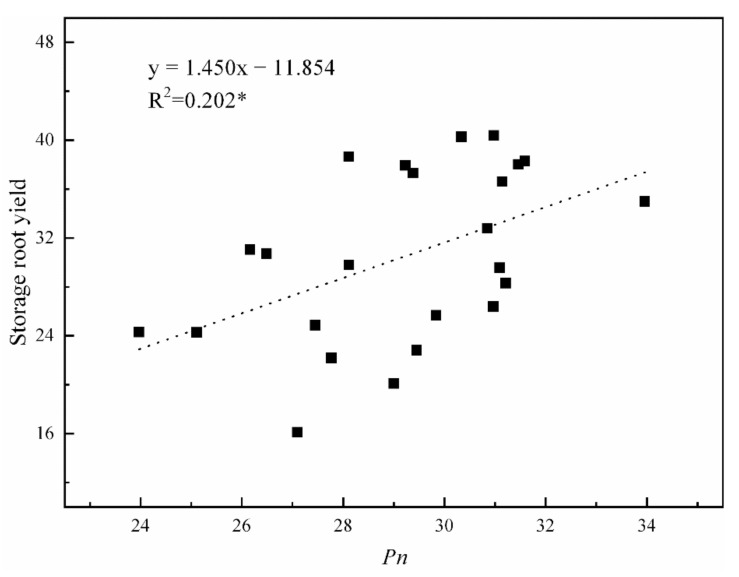
Relationship of the storage root yield (Mg ha^−1^) with *Pn* (μmol CO_2_ m^−2^ s^−1^) for six K_2_O rates in 2017 and 2018. *n* = 24; * is significant at *p* < 0.05.

In this study, the storage root number appeared to be similar between cultivars, while the fresh weight per storage root, as well as storage root yield, were significantly affected by years, cultivars and K_2_O rates (Table 4). Compared to the no-K treated sweet potato, K led to an increased yield. Averaged across DAT for each K_2_O treatment over two years, compared to the no-K treatment, the storage root yield after the 75–300 kg K_2_O ha^−1^ treatments were increased by 13–29% for Xushu 32 and 17–48% for Ningzishu 1, respectively, whereas the storage root yield after the 375 kg K_2_O ha^−1^ treatment was only increased by 26% for Xushu 32 and 45% for Ningzishu 1, respectively. When all data from K_2_O rates were pooled, the storage root yield had a significant positive relationship with *Pn* (Figure 2). In the following study, three K_2_O rates (0, 150 and 300 kg K_2_O ha^−1^) were selected to analyze the changes of the conversion of sucrose-to-starch in the SPSR.

### 2.5. Changes of Sucrose and Starch Content in SPSR

**Figure 3:**
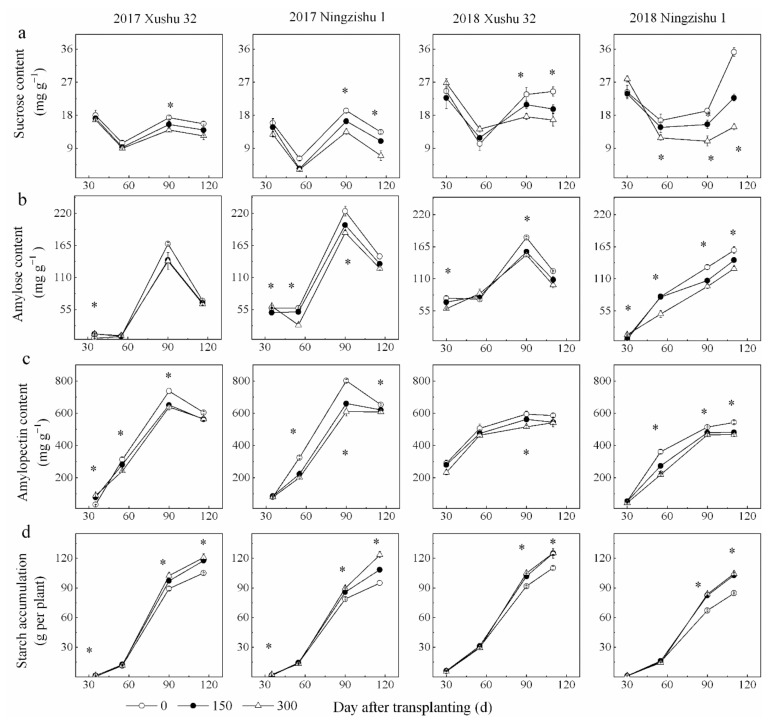
Changes of carbohydrate contents in SPSR under different K_2_O rates in 2017 and 2018. (**a**) Sucrose content; (**b**) amylose content; (**c**) amylopectin content; (**d**) starch accumulation. * is significant among the three K_2_O rates at *p* < 0.05.

Sucrose content in SPSR first declined and then increased with DAT for the three K_2_O rates (Figure 3a), consistent with a previous study [26]. Sucrose content in SPSR decreased with an increasing K_2_O rate and the difference of the sucrose content among the three K_2_O rates increased with DAT. In addition, the decrease range (↓Δ%) of sucrose in SPSR for Ningzhishu 1 (9–15%) was remarkably higher than that of Xushu 32 (17–34%).

The contents of amylose and amylopectin in SPSR first increased and then declined or increased further with DAT for the three K_2_O rates (Figure 3b,c). K application reduced amylose and amylopectin content in SPSR. Compared to no-K treatment, K application decreased the average amylose and amylopectin content across DAT by 9–13% and 6–11% for Xushu 32, respectively, and by 10–23% and 13–19% for Ningzishu 1, respectively.

The trend of total starch accumulation with DAT was similar to that of storge root biomass (Figure 3d and Figure 2b). Compared to the no-K treatment, K application enhanced starch accumulation across DAT by 11–14% and 11–21% for Xushu 32 and Ningzishu 1, respectively.

### 2.6. Enzyme Activities Involved in the Sucrose–Starch Metabolism in SPSR

**Figure 4:**
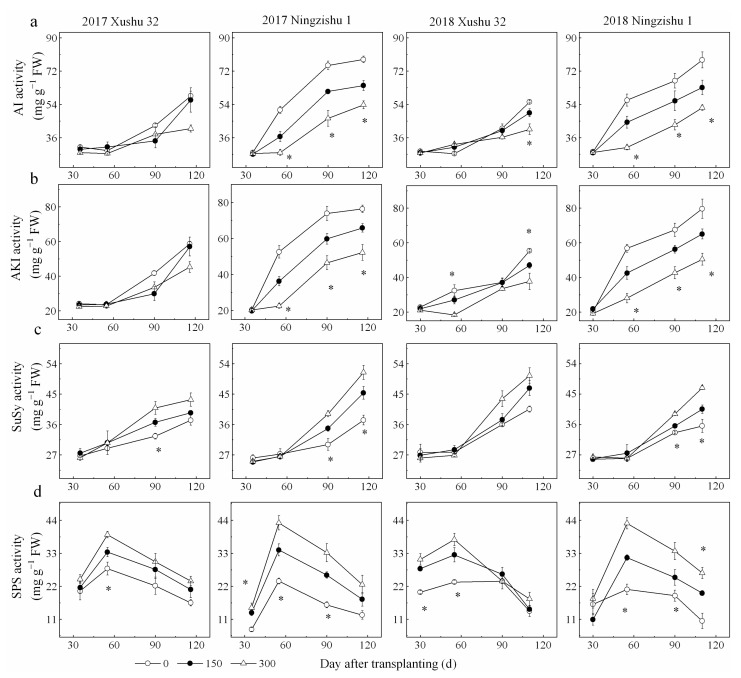
Effect of the K_2_O rates on sucrose-metabolizing enzyme activities in SPSR. (**a**) AI activity; (**b**) AKI activity; (**c**) SuSy activity; (**d**) SPS activity. * is significant among the three K_2_O rates at *p* < 0.05.

The activities of AI, AKI and SuSy in SPSR remained increasing with DAT for the three K_2_O rates (Figure 4a–c). As the K_2_O rates increased, AI and AKI activities were significantly reduced, while SuSy and SPS activities were significantly enhanced (Figure 4).

**Figure 5:**
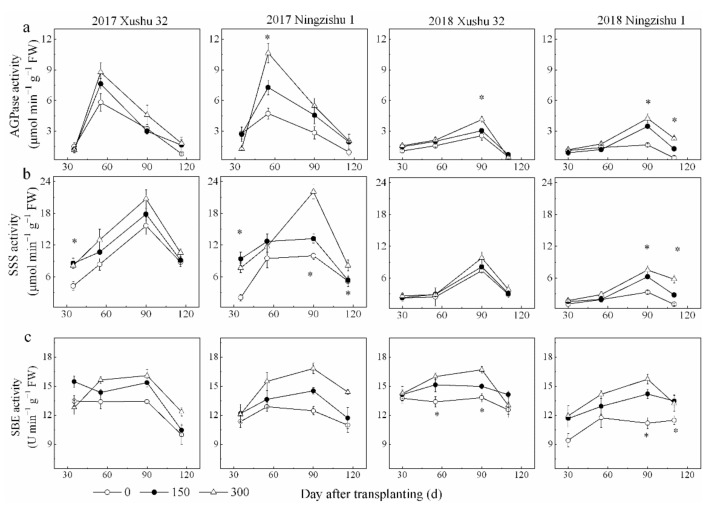
Effect of K_2_O rates on starch-metabolizing enzyme activities in SPSR. (**a**) AGPase activity; (**b**) SSS activity; (**c**) SBE activity. * is significant among the three K_2_O rates at *p* < 0.05.

The pattern of AGPase, SSS and SBE were similar, increasing from 30 DAT, reaching their peak values at 55–90 DAT in 2017 and 90–116 DAT in 2018 and then declining quickly at 120 DAT (Figure 5a–c). Compared to the no-K treatment, K application enhanced the activities of AGPase, SSS and SBE significantly by 19–110%, 13–144% and 9–26%, respectively. 

**Figure 6:**
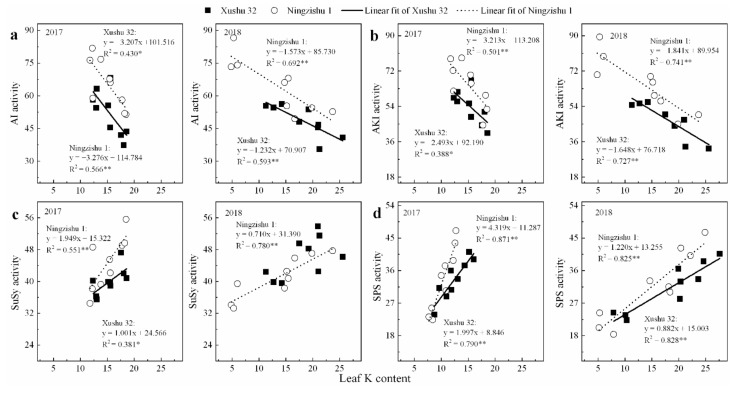
Relationships between the peak values of sucrose-metabolizing enzyme activities in SPSR and leaf K content in 2017 and 2018. (**a**) acid invertase (AI) activity; (**b**) alkaline invertase (AKI) activity; (**c**) SuSy activity; (**d**) SPS activity. The solid and dotted lines represent Xushu 32 and Ningzishu 1, respectively. * and ** are significant among the three K2O rates at *p* < 0.05 and *p* < 0.01, respectively.

**Figure 7:**
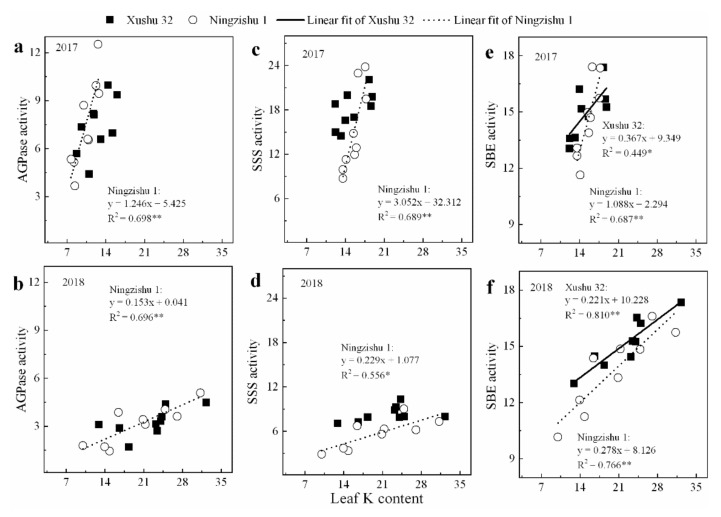
Relationships between the peak values of starch-metabolizing enzyme activities in SPSR and leaf K content in 2017 and 2018. (**a**) AGPase activity in 2017; (**b**) AGPase activity in 2018; (**c**) SSS in 2017; (**d**) SSS in 2018; (**e**) SBE in 2017; (**f**) SBE in 2018. The solid and dotted lines represent Xushu 32 and Ningzishu 1, respectively. * and ** are significant among the three K2O rates at *p* < 0.05 and *p* < 0.01, respectively.

Furthermore, except for SuSy, AGPase and SSS in SPSR for Xushu 32, the peak values of AI and AKI in SPSR for both cultivars were negatively correlated with leaf K content (*p* < 0.05, Figure 6a,b), whereas peak values of SuSy, SPS, AGPase, SSS and SBE were significantly positively correlated with leaf K content (Figure 6c,d and Figure 7a–f).

### 2.7. Genes Transcription Involved in the Sucrose–Starch Metabolism in SPSR

**Figure 8:**
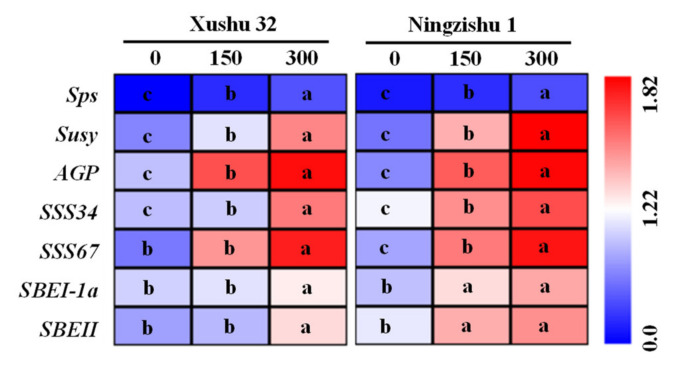
Expression of carbohydrate-metabolizing genes in SPSR under different K_2_O rates in 2018. Blue and red represent decreased and increased expression levels, respectively. Values followed by different letters among the three K_2_O rates are significantly different at *p* < 0.05.

Significant differences were observed in the transcription of *Sps*, *Susy*, *AGP*, *SSS* and *SBE* in SPSR among three K_2_O rates (Figure 8). The transcriptional levels of those genes at 90 DAT were ranked as 300 > 150 > 0 kg K_2_O ha^−1^ among the three K_2_O rates. Moreover, the transcriptional level showed a descending order of *AGP* > *SSS67* > *SSS34* > *Susy* > *SBEI-1a* > *SBEII* > *Sps*.

### 2.8. Relationship Between Enzyme Activities Related to Sucrose and Starch with Starch Accumulation and Storage Root Yield

A correlation analysis of key enzyme activities, involved in sucrose-to-starch conversion, with storage root yield and total starch accumulation showed that SPS, SuSy, AGPase, SSS and SBE in SPSR was significantly positively correlated with the total starch accumulation; AGPase and SSS in SPSR was significantly positively correlated with the yield of storage root (Table 5). 

## 3. Discussion

Previous studies have shown that K can improve crop stress resistance by optimizing gas exchange, stomatal conductance, protein synthesis, enzyme activation and photochemical transport [27,28]. Statistically, at present, about 60% of the total cultivated land area is short of K in China, among which land with a serious shortage of K (available K < 70 mg kg^−1^) occupies 22.6% of the total cultivated land area [29]. The yield and quality decline of SPSR, caused by soil K deficiency, will be increasingly aggravated, which has become one of the important reasons to restrict the production in SPSR. It has been reported that K application of 240–300 kg K_2_O ha^−1^ at basal was given high storage root yields [19], in accordance with the yield of SPSR observed in this study. It was found that the K application of 225–300 kg K_2_O ha^−1^ could enhance the storage root yield by 28–30% and 46–48% for Xushu 32 and Ningzishu 1, respectively, compared to the no-K treatment (Table 5). Therefore, this study further analyzed the physiological mechanisms of K application to improve sweet potato yield. A growth analysis showed that K application significantly affects sweet potato growth, source–sink relation and yield formation.

### 3.1. Plant Growth, Biomass Accumulation and Distribution in SPSR was Improved by Appropriate K Application

As is known, K application could be favorable to plant growth and yield formation, especially in terms of nutrients [21,30]. K application improved sweet potato plant agronomic traits overall [31], showing a longer fast accumulation phase duration (*T*) of plant and SPSR biomass accumulation with a higher growing speed (*V_T_*, Table 2 and Table 3) for both cultivars. Similar results have been observed in cotton (*Gossypium hirsutum* L.) [32]. In terms of biomass accumulation, higher biomass accumulation in SPSR were observed in the K-supply treatments for Xushu 32 and Ningzishu 1 (Table 4), with more than 75% of the biomass accumulation observed in SPSR. In this study, the effect of K application on the yield of SPSR were mainly attributed of the impact on biomass production.

The production of biomass depends on leaf photosynthetic characteristics and sucrose-to-starch conversion in SPSR [26,30,33]. In this study, K application enhanced sweet potato photosynthetic capacity at 35 DAT and 55 DAT, accompanied with an increased storage root weight (Table 4). With the increase of the K_2_O rate, leaf *Pn*, *gs* and *Ci* all increased first and then decreased, and the peak value was between 150–225 kg K_2_O ha^−1^. Previous studies showed that with a treatment of mild K deficiency, the decrease of *Pn* was mainly due to low *gs* [34]. In this study, low *gs* and *Pn* with regards to decreased *Ci* were found in the no-K treatment for both cultivars at 35 DAT, suggesting that stomatal limitation was the dominant factor leading to the decrease of *Pn*. Subsequently, because of the lower *gs* and *Pn* accompanied with increased *Ci*, it seemed that non-stomatal factors played more critical roles in reducing *Pn* in the no-K treatment. These results was consistent with reports on cotton [32] and hickory (*Caya cathayensis* Sarg.) [35]. If the reduction of *Pn* was mainly caused by an increase of *Ls*, then *Ci* must decrease; if the reduction of *Pn* was dominated by an increase in non-stomatal limitations, *Ci* would be increased. In this study, the reduced *Pn* was apparent along with an increase in *Ls* in the no-K treatment (Table 1), but *Ci* increased at 55 DAT, indicating that the reduction of *Pn* was largely due to non-stomatal factors at 55 DAT for Xushu 32 and Ningzishu 1. Therefore, the conclusion obtained from the *Ls* value was in accordance with the above interpretation and analysis in Table 1.

### 3.2. Appropriate K Application Is Beneficial to Sucrose-to-Starch Conversion in SPSR

Starch is the major form of fixed carbon in SPSR, about 50–80% of SPSR’s dry biomass [4,5]. Rational fertilization is of great importance to obtain a high starch yield. In this study, K application enhanced starch accumulation in SPSR compared to the no-K treatment (Figure 3d). However, the content of starch in SPSR, as the sum of amylose and amylopectin (Figure 3b,c), was decreased by K application, probably because of the dilution effect of protein [36]. Starch synthesis in plastids is closely related to the hydrolysis of sucrose in cytoplasm [17], which involves many enzymes and their coding genes that convert sucrose to starch during SPSR development. 

The local content of sucrose in SPSR varies with the enzymes involved in the synthesis and breakdown of sucrose, which further determines the sucrose unloading from phloem [37]. Additionally, the decomposition products of sucrose, glucose and fructose could provide essential substrates to biosynthesis starch [38]. The main enzymes are SPS for sucrose synthesis, and AI, AKI and SuSy (cleavage) for sucrose decomposition [39]. A reduced sucrose content in SPSR was found with the increase of K_2_O rates (Figure 3a), possibly causing more sucrose to break down to produce more substrates to synthesize starch. It was reported that in tuber crops, the main component of sucrose decomposition are invertase in the early stage of root formation and SuSy (cleavage) in the late stage [15]. Compared to the SPSR of the no-K treatment, the K-treated SPSR had higher SuSy (cleavage) and lower AI; moreover, AKI decreased, indicating that K application increased sucrose decomposition by increasing the transcriptional level and enzymatic activity of SuSy. Surprisingly, K application not only brought in higher SuSy activity for cleavage but also higher SPS activity in SPSR (Figure 4c,d). In comparison, K application also improved the transcriptional level of *Susy* relative to that of *Sps* in the K-treated SPSR (Figure 8), and the SuSy activity was much higher than the SPS activity. Moreover, SuSy (cleavage) activity was significantly positively correlated with starch accumulation and storage root yield (Table 5). The results were confirmed by the higher ABA in SPSR at the stage of storage root expansion [40], which could inhibit SuSy and enhance the synthesis of starch [41]. Therefore, SuSy activity for cleaving in the K-treated SPSR considerably exceeded the SPS activity for synthesis, thus promoting the decomposition of sucrose and providing sufficient substrates for the synthesis of starch.

**Figure 9:**
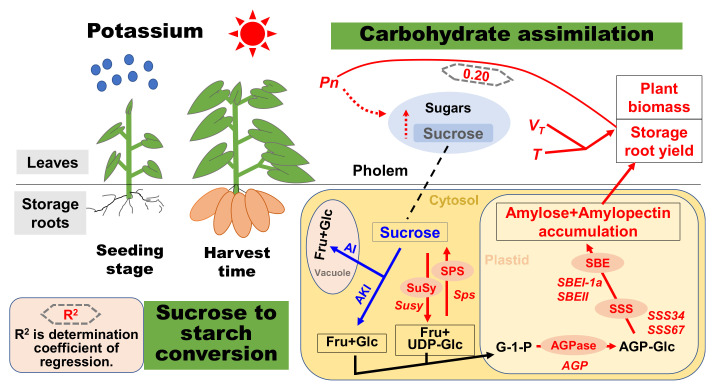
Increases in metabolite concentrations and enzyme activities in the K-treated sweet potato are shown in red, and decreases are shown in blue. *T* is the period of fast biomass accumulation phase; *V_T_* is the average biomass accumulation rate during the fast biomass accumulation duration.

AGPase, SSS and SBE play critical roles in starch synthesis [17]. AGPase, a speed-limiting enzyme in starch synthesis, catalyzes ADP-glucose synthesis, used for the synthesis of starch polymers [16]. In tuber root, higher sucrose from phloem unloading could up-regulate the transcriptional level of *AGP* [42]. K application could produce more carbon from leaf to SPSR during SPSR development (Figure 2), thus up-regulating *AGP* (Figure 8) and improving the AGPase activity in the K_2_O-treated SPSR (Figure 7a). SSS and SBE play critical roles in the synthesis of amylose and amylopectin [16,17]. Appropriately increasing the fertilizer to soil could improve the enzyme activities and transcriptional levels of SSS and SBE, thus leading to a high starch yield in tuber root and grain [43,44], consistent with our study, showing that the activities of SSS and SBE and transcriptional levels of *SSS34*, *SSS67*, *SBEI-1a* and *SBEII* in SPSR were increased by K application. In this study, starch accumulation, as well as the yield in SPSR, was significantly positively correlated with AGPase and SSS (Table 5), in agreement with previous studies [19,26]. In this study, the most noteworthy is that the ↑△AGPase and ↑△SSS were more than ↑△SBE. Additionally, the transcriptional level of *AGP* was higher than those of *Susy*, *SSS34* and *SSS67* in SPSR (Figure 8). The results indicated that K application promoted the synthesis of starch and storage root yield through improving the activities and transcriptional levels of AGPase and SSS, and AGPase is the speed-limiting enzyme in SPSR for the synthesis of starch in the K-treated SPSR. Based on the results and discussion above, we propose a schematic overview of K application on sweet potato growth and the conversion of sucrose to starch in SPSR (Figure 9).

The two cultivars, Xushu 32 and Ningzishu 1, exhibited similar patterns in carbon assimilation following K application. However, Ningzishu 1 showed a larger change for sucrose–starch metabolism in the K-treated sweet potato compared to Xushu 32. Based on the analysis of this study, in the process of sucrose–starch metabolism, SuSy plays a critical role in sucrose decomposition, and AGPase and SSS are the important roles in starch synthesis. In this study, for the two cultivars, there were different sensitivities to K in SuSy, AGPase and SSS activities in SPSR. The average SuSy increased by 6–12% in the K-treated SPSR (150 and/or 300 kg K_2_O ha^−1^) compared to the no-K treated SPSR for Xushu 32, and by 7–18% for Ningzishu 1. The average AGPase and SSS increased by 19–46% and 13–38% for Xushu 32, respectively, and by 46–110% and 52–144% for Ningzishu 1, respectively. In addition, for Ningzishu 1, there were linear relationships between these peak values of SuSy, AGPase and SSS and leaf K content, whereas no correlation was found between these peak values for Xushu 32 and leaf K content (Figure 7), suggesting that Ningzishu 1 was much more influenced by K than Xushu 32. Therefore, K application enhanced the conversion of sucrose to starch more in Ningzishu 1 compared with Xushu 32. 

## 4. Materials and Methods

### 4.1. Experimental Design

In the field experiments, two sweet potato cultivars, Ningzishu 1 (low-K sensitive) and Xushu 32 (low-K-tolerant) [24,25], were gown in 2017 and 2018 at the Xuzhou Academy of Agricultural Sciences, Xuzhou, Jiangsu, China (117°29′ E, 34°29′ N). The soil at the experimental site was loamy and yellow fluve-aquic (pH 7.8). In 20-cm deep soil, in 2017, the content of organic matter was 16.8 g kg^−1^, the content of hydrolysable N was 91 mg kg^−1^ and the content of available K and P were 71 and 23 mg kg^−1^, respectively; in 2018, the content of organic matter was 16.2 g kg^−1^, the content of hydrolysable N was 94 mg kg^−1^ and the content of available K and P were 85 and 20 mg kg^−1^, respectively.

The experiment was designed with three completely random replications. A total of 150 kg ha^−1^ N (urea) and 75 kg ha^−1^ P_2_O_5_ (calcium superphosphate) was applied as a uniform fertilizer application. The treatments consisted of six K_2_O rates: 0, 75, 150, 225, 300 and 375 kg K_2_O ha^−1^ using potassium sulphate. The control was treatment of 0 kg K_2_O ha^−1^. All these fertilizer applications were applied as base fertilizer in each treatment. The row spacing × plant spacing was 0.85 m × 0.25 m, and the final planting density was 46,500 plants ha^−1^.

### 4.2. Sampling and Processing

Three continuous sweet potato plants were collected from the central row of each treatment. Sweet potato biomass was determined at different growth stages from 35 days after transplanting (DAT) to 120 DAT in 2017 and from 30 to 120 DAT in 2018. The collected plants were divided into stem + vine, leaf, root and storage root for each treatment. Subsequently, the samples were first oven-dried at 105 °C for half an hour and then at 65 °C until the weight was constant.

Simultaneously, the fresh storage roots (diameters > 0.5 cm) were collected and washed with dd H_2_O. Each fresh SPSR was divided into two parts from the middle of SPSR, and cut into slices. One part was immediately frozen in liquid N_2_ and saved in a refrigerator (−80 °C) to measure the sucrose–starch metabolizing enzyme activities and their gene transcriptions. The other was oven-dried for carbohydrate analysis.

At the mature stage, the SPSR per plant was collected by hand for each treatment. The number of storage root per plant, fresh weight per storage root and storage root yield, as yield components, were counted and recorded.

### 4.3. Leaf Photosynthesis Parameters

At 35 DAT and 55 DAT, *Pn*, stomatal conductance (*gs*) and intercellular CO_2_ concentration (*Ci*) in functional leaves (the third leaf from the top) were measured by a Li-6400 (Li-COR Inc., NE, USA) on a sunny day at 9:00–11:00 am. Measurements were made using under 1000 μmol m^−2^ s^−1^ light intensity with 400 μmol mol^−1^ CO_2_ and (65 ± 5)% relative humidity.

### 4.4. Sucrose and Starch Analysis

Dried storage root tissues were ground by a ball mill instrument. A total of 50 mg of the ground sample was put into a tube of 2 mL and extracted twice using 80% ethanol (500 μL) at 80 °C. Thereafter, the extracting solution was diluted into 1.8 mL and mixed for the determination of sucrose according to previously described protocols [14]. 

The content of amylose and amylopectin were determined following previous methods with some modifications [16]. Briefly, a 50-mg storage root tissue was weighed into a 15 mL centrifuge tube, followed by adding 250 μL of 100% ethanol and 1.25 mL NaOH (1 M), and the centrifuge tube was cooled after boiling for 15 min, after which 11 mL dd H_2_O was added and the sample was vigorously mixed for subsequent measurements. Then, 0.3 mL of extract was added into another centrifuge tube containing 50 μL acetic acid (1 M), 50 μL iodine solution (0.2 g I_2_ and 2.0 g KI in 100 mL dd H_2_O) and 2.1 mL of dd H_2_O, mixed vigorously and let stand for 20 min. The OD values were recorded at 630 nm and 460 nm for amylose, and at 550 nm and 740 nm for amylopectin using a microplate reader.

### 4.5. Sucrose–Starch Metabolizing Enzymatic Extraction and Analysis

A total of 0.1 g of the frozen storage root sample was homogenized with 1.8 mL cool extraction buffer. Tris-HCl (pH 7.5, 100 mM), MgCl_2_ (8 mM), EDTA (2 mM), glycerin (12.5%, *v*/*v*) and β-mercaptoethanol (50 mM) were contained in the extraction buffer. The supernatant after centrifugation at 10,000× *g* at 4 °C was collected to analyze the activities of enzymes (SPS, SuSy, AI, AKI, AGPase, SSS, and SBE) in sucrose–starch metabolism.

SPS activity was measured using the UDP-Glucose method and SuSy activity in the direction of sucrose decomposition was determined using the UDP method, as previous studies described [45,46]. The activities of AI and AKI were analyzed using the 3,5-dinitro salicylic acid (DNS) method, by sucrose (1 M) in acetic acid–NaOH (200 mM, pH 5.0) for AI or sodium acetate–acetic acid (100 mM, pH 7.5) for AKI. 

AGPase and SSS were assayed by measurement of the formation of NADPH using a microplate reader according to a previous study with some modifications [47,48]. The reaction mixture I for AGPase contained Hepes-NaOH (100 mM, pH 7.4), ADPG (1.2 mM), sodium pyrophosphate (3 mM), MgCl_2_ (5 mM) and DTT (4 mM). The reaction mixture II for AGPase contained NADP (10 mM), P-glucomutase (0.4 U) and glucose 6-phosphate dehydrogenase (0.4 U). The reaction mixture I for SSS contained Hepes-NaOH (50 mM, pH 7.4), ADPG (1.6 mM), amylopectin (0.1 mg) and DTT (15 mM). The reaction mixture II for SSS contained Hepes-NaOH (50 mM, pH 7.4), phosphoenolpyruvic acid (4 mM), KCl (200 mM), MgCl_2_ (10 mM) and pyruvate kinase (1.2 U). The reaction mixture III for SSS contained Hepes-NaOH (50 mM, pH 7.4), glucose (10 mM), MgCl_2_ (20 mM), NADP (2 mM), hexokinase (1.4 U) and glucose 6-phosphate dehydrogenase (0.35 U). SBE (EC 2.4.1.18) was determined as described previously [47,48]. 

The units of enzyme activity were recorded as U min^−1^ g^−1^ FW for SBE, μmol min^−1^ g^−1^ FW for AGPase and SSS and mg g^−1^ FW for others.

### 4.6. RNA Extraction and Gene Transcriptional Analysis in SPSR

Total RNA from SPSR were extracted according to the manufacturer’s instructions of RNA-prep pure plant kit (DP441-50T, Tiangen Biotech, Beijing, China). Two μg of Total RNA was reverse transcribed using a Prime Script RT reagent cDNA kit (TaKara, Dalian, China). The primers of related genes are shown in Supplementary Table S1. *GAPDH* was used as a reference gene, and the relative transcriptional levels of *Sps*, *Susy*, *AGP*, *SSS34, SSS67, SBEI-1a* and *SBEII* were calculated using the 2^−ΔΔCT^ method [49,50].

### 4.7. Weather Data and Statistical Analysis

Weather data in Xuzhou were obtained from the National Meteorological Information Center. The mean daily temperature and total sunshine hours during the sweet potato growth stage (from June to October) was lower in 2017 than in 2018 (Table 6). However, the total precipitation in August was higher in 2018 than in 2017. 

Origin and Microsoft Excel 2019 were used for data analysis. SPSS was used for the analysis of variance through using the difference between the average values identified by the Tukey test (HSD, *p* < 0.05) and for the regression analysis. The stomatal limitation value (*Ls*) = 1 − *Ci*/*Ca* (*Ca* is the external CO_2_ concentration) is used to represent the stomatal limit value. The limitation of non-stomatal factors (Non-*Ls*) is usually expressed as a *Ci*/*gs* ratio.

Crop biomass dynamic accumulation is extensively described by the following logistic model:

(1) $$y=\frac{y_{max}}{1+ae^{-b\times DAT}}$$

where y (g per plant) is sweet potato biomass, *DAT* (d) is days after transplanting, *y_max_* (g) is the maximum biomass and a and b are parameters.

From Equation (1):

(2) $$DAT_{0}=\frac{a}{b},\;DAT_{1}=-\frac{1}{b}ln\frac{2+\sqrt{3}}{a},\;DAT_{2}=-\frac{1}{b}ln\frac{2-\sqrt{3}}{a},\;DAT_{m}=\frac{lna}{b}$$

The accumulation of sweet potato biomass was at the maximum rate when *DAT* = *DAT*_0_:

(3) $$V_{max}=\frac{b\;\times \;y_{max}}{4}\;,\;V_{T}=\frac{y_{2}\;-\;y_{1}}{DAT_{2}\;-\;DAT_{1}}$$

where *DAT_1_* and *DAT_2_* are the DAT when biomass rapid increase started and when the rapid increase terminated, respectively, *DAT_m_* is the DAT when the rate of biomass accumulation reached maximum, *T* (d) = *DAT*_2_ − *DAT*_1_ is the rapid biomass increase period, *V_max_* is the maximal rate of biomass accumulation and *V_T_* is the average rate of biomass accumulation.

## 5. Conclusions

1) Compared to the no-K treatment, sweet potato yield was remarkably improved by K application, although treatments at high levels of K, i.e., 375 kg K_2_O ha^−1^, significantly decreased storage root yield. K application enhanced biomass accumulation of plant and storage root yield by promoting *V_T_* and *T*.
2) K application enhanced the photosynthetic capacity of sweet potato at 35 DAT and 55 DAT, accompanied with an increased storage root weight. The reduction of *Pn* induced by K deficiency was mainly caused by stomatal limitation at 35 DAT and by non-stomatal factors at 55 DAT.
3) K application promoted the starch accumulation mainly by improving the activities of SuSy, AGPase and SSS, and these genes’ transcriptions, and AGPase is the speed-limiting enzyme for the synthesis of starch in SPSR of the K-treated treatments.
4) Future research is required to determine the correlation of K ^+^ transport activity in root and K tolerance in sweet potato, in order to breed low-K-tolerant sweet potato cultivars in the future.

## Figures and Tables

**Table 1:** Effect of K_2_O rates on photosynthetic parameters, stomatal limitation and non-stomatal limitation in the functional leaf of sweet potato. Each value represents the mean of four replications.

Cultivar	K_2_O Rate (kg ha^−1^)	*Pn* (μmol CO_2_ m^−2^ s^−1^)		*gs* (mol H_2_O m^−2^ s^−1^)		*Ci* (μmol CO_2_ mol^−1^)		Non-*Ls*		Ls	
		35 DAT	55 DAT	35 DAT	55 DAT	35 DAT	55 DAT	35 DAT	55 DAT	35 DAT	55 DAT
2017											
Xushu 32	0	30.85 ab	28.3 b	0.63 d	0.72 b	321.9 bc	339.9 a	514.6 a	471.9 ab	0.16 a	0.12 bc
	75	31.14 ab	29.16 ab	0.69 cd	0.77 ab	325.2 b	330.5 bc	473.2 ab	431.8 bc	0.13 b	0.11 abc
	150	31.59 a	29.13 ab	0.73 bc	0.89 a	328.4 a	327.9 c	449.6 bc	366.9 c	0.14 b	0.1 c
	225	30.98 ab	30.29 a	0.82 a	0.79 ab	323.2 bc	332.3 bc	397.6 c	435.5 abc	0.16 a	0.11 abc
	300	30.34 b	29.85 a	0.78 ab	0.69 b	322.3 bc	335.2 ab	414.3 c	492.6 ab	0.14 ab	0.11 ab
	375	28.11 c	29.95 a	0.73 bc	0.63 b	320.7 c	332.7 bc	442.8 bc	533 a	0.15 ab	0.11 a
CV across K_2_ O rate (%)		3.71	2.25	8.25	10.91	0.78	1.13	8.52	11.51	7.65	4.62
Ningzishu 1	0	30.97 b	27.94 c	0.69 bc	0.49 b	319.6 c	338.7 b	472.8 ab	699.7 a	0.17 a	0.12 ab
	75	31.09 b	31.24 a	0.8 a	0.79 a	326.3 b	330 b	407.6 b	422 b	0.15 b	0.11 bc
	150	33.96 a	30.79 ab	0.83 a	0.8 a	332.1 a	327.5 b	402.5 b	424.4 b	0.14 b	0.11 bc
	225	31.46 b	30.14 ab	0.77 ab	0.75 a	325 b	332.6 b	422.1 b	448.1 b	0.15 ab	0.1 c
	300	29.23 c	29.5 b	0.65 c	0.69 a	325.1 b	340.3 a	509 a	502.7 b	0.14 b	0.1 c
	375	29.38 c	30.14 ab	0.63 c	0.48 b	318.1 c	330.8 b	505.2 a	703.7 a	0.13 b	0.13 a
CV across K_2_O rate (%)		5.05	3.52	10.32	19.82	1.41	1.26	9.79	22.13	9.00	9.28
Analysis of variance	Cultivar (C)	*	*	ns	**	ns	ns	ns	**	ns	ns
	K_2_O rate (K)	**	**	**	**	**	**	**	**	**	**
	C × K	**	*	**	ns	ns	*	**	*	ns	**
2018											
Xushu 32	0	27.77 bc	18.96 b	0.76 ab	0.69 bc	297.69 a	309.6 ab	399.4 abc	455.9 ab	0.18 b	0.18 a
	75	29.84 ab	19.15 b	0.76 ab	0.75 abc	299.69 a	300.8 ab	396.3 bc	411.3 abc	0.17 ab	0.17 a
	150	31.21 a	21.85 a	0.83 a	0.84 a	301.2 a	294.5 b	365.2 c	353.4 c	0.17 a	0.19 a
	225	28.12 bc	21.19 a	0.64 b	0.79 ab	304 a	296.5 ab	477.8 ab	376.1 bc	0.16 ab	0.16 a
	300	26.16 c	21.91 a	0.62 b	0.7 bc	301.1 a	294.4 b	500.3 a	426.2 abc	0.17 ab	0.19 a
	375	26.49 c	20.46 ab	0.6 b	0.65 c	300 a	294.3 b	500.7 a	458.8 a	0.16 ab	0.19 a
CV across K_2_ O rate (%)		6.30	5.75	12.14	8.93	0.92	1.85	12.02	9.37	4.80	8.17
Ningzishu 1	0	27.1 b	20.86 c	0.47 bc	0.46 b	281.75 c	292.7 a	607.6 ab	646.5 a	0.23 a	0.25 b
	75	29 ab	24.98 ab	0.5 bc	0.55 ab	293.73 bc	283.1 ab	589.3 ab	520 ab	0.18 b	0.24 b
	150	29.45 a	26.05 a	0.72 a	0.61 a	300.5 ab	283.7 ab	420.3 c	466.6 b	0.17 bc	0.23 b
	225	27.45 ab	22.67 bc	0.57 b	0.55 ab	310 a	291.5 ab	547.1 b	548.4 ab	0.14 c	0.21 b
	300	25.11 c	21.99 c	0.45 c	0.45 b	305.6 ab	279.6 bc	689.2 a	633.2 a	0.18 b	0.29 a
	375	23.96 c	22.53 bc	0.48 bc	0.44 b	297.8 ab	270.3 c	630.5 ab	630 a	0.16 bc	0.22b
CV across K_2_O rate (%)		7.25	7.66	17.41	12.61	3.03	2.65	14.39	11.69	15.49	11.68
Analysis of variance	Cultivar (C)	**	**	**	**	ns	**	**	**	*	**
	K_2_O rate (K)	**	**	**	**	*	**	**	**	**	**
	C × K	ns	*	ns	ns	**	ns	ns	ns	**	ns

Non-*Ls*, non-stomatal limitation; *Ls*, stomatal limitation; CV, coefficient of variation. Values followed by a different letter among the six different K_2_O rates for each cultivar are significantly different at a *p* < 0.05 probability level. ns, non-significant; * and **, significant at the *p* < 0.05 and 0.01 probability levels, respectively.

**Table 2:** Effects of K_2_O rates on the eigenvalues of sweet potato biomass accumulation in 2017 and 2018.

K_2_O Rate (kg ha^−1^)	2017					2018				
	Equations	r	*Tm* (d)	*T* (d)	*V_T_* (g d^−1^)	Equations	r	*Tm*^a^ (d)	*T* (d)	*V_T_* (g d^−1^)
Xushu 32										
0	y = 157.10/(1 + 89.53 × 10^−0.057DAT^)	0.975 **	78.85	46.21	1.96	y = 165.83/(1 + 50.42 × 10^−0.055DAT^)	0.991 **	71.28	47.89	2.00
75	y = 166.40/(1 + 72.08 × 10^−0.053DAT^)	0.983 **	80.71	49.69	1.93	y = 187.80/(1 + 50.07 × 10^−0.053DAT^)	0.979 **	73.84	49.69	2.18
150	y = 222.81/(1 + 89.82 × 10^−0.053DAT^)	0.997 **	84.87	49.69	2.59	y = 213.78/(1 + 53.82 × 10^−0.053DAT^)	0.997 **	75.20	49.69	2.48
225	y = 213.52/(1 + 89.32 × 10^−0.053DAT^)	0.995 **	84.76	49.69	2.48	y = 212.18/(1 + 50.35 × 10^−0.054DAT^)	0.993 **	72.57	48.77	2.51
300	y = 224.31/(1 + 94.65 × 10^−0.055DAT^)	0.996 **	82.73	47.89	2.70	y = 231.64/(1 + 54.02 × 10^−0.053DAT^)	0.999 **	75.27	49.69	2.69
375	y = 199.42/(1 + 94.625 × 10^−0.059DAT^)	0.980 **	77.12	44.64	2.58	y = 221.99/(1 + 54.706 × 10^−0.056DAT^)	0.999 **	71.46	47.03	2.72
Ningzishu 1										
0	y = 125.658/(1 + 68.704 × 10^−0.059DAT^)	0.972 **	71.69	44.64	1.63	y = 139.612/(1 + 51.984 × 10^−0.055DAT^)	0.987 **	71.84	47.89	1.68
75	y = 145.733/(1 + 63.953 × 10^−0.056DAT^)	0.959 **	74.25	47.03	1.79	y = 182.133/(1 + 55.6 × 10^−0.053DAT^)	0.996 **	75.81	49.69	2.12
150	y = 161.896/(1 + 75.734 × 10^−0.055DAT^)	0.981 **	78.68	47.89	1.95	y = 198.041/(1 + 53.901 × 10^−0.053DAT^)	0.990 **	75.23	49.69	2.30
225	y = 163.704/(1 + 64.517 × 10^−0.056DAT^)	0.932 **	74.41	47.03	2.01	y = 215.008/(1 + 39.283 × 10^−0.047DAT^)	0.997 **	78.10	56.04	2.22
300	y = 178.201/(1 + 52.325 × 10^−0.051DAT^)	0.950 **	77.60	51.64	1.99	y = 199.695/(1 + 49.977 × 10^−0.054DAT^)	0.999 **	72.44	48.77	2.36
375	y = 175.247/(1 + 43.939 × 10^−0.054DAT^)	0.943 **	70.05	48.77	2.07	y = 200.598/(1 + 46.646 × 10^−0.054DAT^)	0.999 **	71.16	48.77	2.37

^a^*T_m_* is the DAT when biomass accumulation reached the maximum rate of increase; *T* is the period of fast biomass accumulation phase; *V_T_* is the average biomass accumulation rate during the fast biomass accumulation duration. ** means significant at the *p* < 0.01 probability levels.

**Table 3:** Effects of K_2_O rates on the eigenvalues of tuberous biomass accumulation in 2017 and 2018.

K_2_O Rate (kg ha^−1^)	2017					2018				
	Equations	r	*Tm* (d)	*T* (d)	*V_T_* (g d^−1^)	Equations	r	*Tm* ^a^ (d)	*T* (d)	*V_T_* (g d^−1^)
Xushu 32										
0	y = 656.00/(1 + 894.30 × 10^−0.087DAT^)	0.985 **	78.12	30.27	12.51	y = 485.20/(1 + 4269.72 × 10^−0.101DAT^)	0.998 **	82.77	26.08	10.74
75	y = 778.91/(1 + 863.84 × 10^−0.085DAT^)	0.981 **	79.55	30.98	14.51	y = 543.64/(1 + 2054.11 × 10^−0.091DAT^)	0.989 **	83.82	28.94	10.84
150	y = 808.98/(1 + 562.90 × 10^−0.08DAT^)	0.996 **	79.16	32.92	14.19	y = 597.03/(1 + 1234.96 × 10^−0.085DAT^)	0.991 **	83.75	30.98	11.12
225	y = 867.86/(1 + 590.41 × 10^−0.079DAT^)	0.988 **	80.77	33.34	15.03	y = 638.24/(1 + 799.19 × 10^−0.079DAT^)	0.991 **	84.60	33.34	11.05
300	y = 899.69/(1 + 562.90 × 10^−0.080DAT^)	0.998 **	83.77	36.08	14.40	y = 691.50/(1 + 663.53 × 10^−0.078DAT^)	0.997 **	83.30	33.77	11.82
375	y = 813.28/(1 + 471.90 × 10^−0.079DAT^)	0.996 **	77.93	33.34	14.08	y = 677.96/(1 + 581.241 × 10^−0.08DAT^)	0.999 **	79.56	32.92	11.89
Ningzishu 1										
0	y = 518.40/(1 + 2960.94 × 10^−0.095DAT^)	0.974 **	84.14	27.72	10.80	y = 327.70/(1 + 3726.37 × 10^−0.102DAT^)	0.995 **	80.62	25.82	7.33
75	y = 656.52/(1 + 1830.32 × 10^−0.085DAT^)	0.998 **	88.38	30.98	12.23	y = 415.73/(1 + 1728.92 × 10^−0.092DAT^)	0.993 **	81.04	28.63	8.38
150	y = 761.77/(1 + 1611.56 × 10^−0.082DAT^)	0.982 **	90.06	32.12	13.69	y = 495.31/(1 + 672.00 × 10^−0.08DAT^)	0.997 **	81.38	32.92	8.69
225	y = 816.70/(1 + 1243.14 × 10^−0.081DAT^)	0.990 **	87.97	32.51	14.50	y = 542.14/(1 + 422.79 × 10^−0.076DAT^)	0.999 **	79.56	34.65	9.03
300	y = 805.13/(1 + 738.72 × 10^−0.075DAT^)	0.997 **	88.07	35.12	13.24	y = 510.95/(1 + 1145.07 × 10^−0.094DAT^)	0.998 **	74.93	28.02	10.53
375	y = 794.73/(1 + 1442.72 × 10^−0.085DAT^)	0.996 **	85.58	30.98	14.81	y = 501.24/(1 + 1165.00 × 10^−0.094DAT^)	0.999 **	75.11	28.02	10.33

^a^*T_m_* is the DAT when biomass accumulation reached the maximum rate of increase; *T* is the period of fast biomass accumulation phase; *V_T_* is the average biomass accumulation rate during the fast biomass accumulation duration. ** means significant at the *p* < 0.01 probability levels.

**Table 4:** Yield and its components of sweet potato as affected by K_2_O rates in 2017 and 2018.

K_2_O Rate (kg K_2_O ha^−1^)	Storage Root Number (No. Plant^−1^)		Fresh Weight Per Storage Root (g)		Storage Root Yield (Mg ha^−1^)		Plant Biomass (g Per Plant)		The Biomass Rate of Storage Root/Whole Plant	
	2017	2018	2017	2018	2017	2018	2017	2018	2017	2018
Xushu 32										
0	3.67 a ^a^	3.33 a	163.9 e	143.1 abc	32.79 c	22.18 c	155.66 c	155.3 c	0.78 a	0.80 c
75	4.33 a	4.33 a	181.7 d	127.4 c	36.61 b	25.67 bc	160.87 bc	170.3 bc	0.82 a	0.83 abc
150	4.33 a	4.33 a	190.1 c	140.5 bc	38.3 ab	28.30 ab	187.53 a	187.4 ab	0.87 a	0.85 ab
225	4.33 a	4.00 a	200.5 b	160.2 ab	40.4 a	29.79 ab	181.88 ab	178.3 abc	0.87 a	0.87 a
300	4.33 a	4.33 a	206.8 a	154.1 ab	40.28 a	31.05 a	193.79 a	196.9 ab	0.88 a	0.87 a
375	4.00 a	4.00 a	207.8 a	165.2 a	38.65 ab	30.72 ab	184.28 ab	202.1 a	0.88 a	0.82 bc
Ningzishu 1										
0	4.00 a	2.33 b	141.9 c	148.5 a	26.40 b	16.11 b	118.71 d	120.3 c	0.77 b	0.71 c
75	4.33 a	3.33 ab	162.2 b	129.7 c	29.58 b	20.10 ab	136.27 cd	154.1 b	0.84 a	0.74 bc
150	4.33 a	3.67 a	189.0 a	132.6 bc	34.99 a	22.82 a	143.78 bc	165.0 ab	0.85 a	0.81 ab
225	5.00 a	4.00 a	183.5 a	126.2 c	38.02 a	24.87 a	158.34 abc	176.2 a	0.86 a	0.83 ab
300	3.67 a	3.67 a	195.3 a	142.4 ab	37.95 a	24.27 a	165.03 ab	175.9 a	0.87 a	0.85 a
375	4.00 a	3.67 a	192.3 a	142.5 ab	37.31 a	24.29 a	167.83 a	177.0 a	0.87 a	0.85 a
Significance of factors										
Year	6.594 *		360.157 **		402.395 **		9.863 **		5.821 *	
Cultivar (C)	ns		34.289 **		78.879 **		78.563 **		6.179 *	
K_2_O rate (K)	2.637 *		19.075 **		29.349 **		27.047 **		10.001 **	
Y × C	ns		ns		ns		ns		ns	
Y × K	ns		10.401 **		ns		ns		ns	
C × K	ns		ns		ns		ns		ns	
Y × C × K	ns		2.665 *		ns		ns		ns	

^a^ Values followed by different letters within a column are significantly different at *p* < 0.05. * and ** are significant at *p* < 0.05 and *p* < 0.01, respectively. ns means no significance.

**Table 5:** Correlation coefficients among sweet potato yield, starch accumulation and sucrose–starch metabolizing enzymes for three different K_2_O rates. *n* = 12, R_0.05_ = 0.5760, R_0.01_ = 0.7079.

Correlation with	SPS	SuSy	AGPase	SSS	SBE
Starch accumulation	0.5833 *^a^	0.7208 **	0.5881 *	0.5779 *	0.7665 **
Storage root yield	ns	ns	0.7992 **	0.7780 **	ns

^a^ * and ** are significant at *p* < 0.05 and *p* < 0.01, respectively. ns means no significance.

**Table 6:** Meteorological data during the sweet potato growing season in 2017 and 2018.

Meteorological Data	Year	June	July	August	September	October	Total
Mean daily temperature (°C)	2017	25.8	29.9	27.7	23.1	15.4	24.4
	2018	27.4	28.8	28.6	22.8	16.1	24.7
Total sunshine hours (h)	2017	265.2	241.2	210.3	191.8	157.8	1066.3
	2018	282.8	242.4	225.3	212.2	238.0	1200.7
Total precipitation (mm)	2017	92.1	279.9	105.1	101.6	75.0	653.7
	2018	48.7	248.5	362.3	62.6	0.2	722.2

## Data Availability

The data used to support the findings of this study are included in the article.

## Supplementary Materials

The following are available online at https://www.mdpi.com/article/10.3390/ijms22094826/s1. Table S1. Specific primers for gene amplification.
