# Supplementary material for: Potassium Fertilization Stimulates Sucrose-to-Starch Conversion and Root Formation in Sweet Potato (Ipomoea batatas (L.) Lam.)
Source: Int J Mol Sci. 2021 May 1;22(9):4826. doi: 10.3390/ijms22094826 (PMC8125193; doi:10.3390/ijms22094826)
Supplement: Supplementary file 1 [file ijms-22-04826-s001.zip › ijms-1205304-supplementary.pdf]

Supplementary Materials

Table S1. Specific primers for gene amplification.

Target gene	Forward primer (5′→3′)	Reverse primer (5′→3′)
Sps	TCTGAAGATTTCTCGGATGA	AGTAAGGAGCATAGGCACA
Susy	CTTGAGATTCGTCGCTACCTT	CTGAACCCTCCCTTCATCTTAC
AGP	GCAGACTTGTCTAGATCCTGATG	CCGCTTCTTTGTGAGAGGATAG
SSS34	GACTGTGGGATCTACTGAAAGG	TTGCTGGCTCCTGAGAATTTA
SSS67	GGAATGGCAAGCAGGAACTA	CCTCCAAGAACATCTCCAAGTC
SBEI-1a	CCTTCTCGTGGGTCTTTCATAC	ACCAGTAGTGCATGGTGAAG
SBEII	CCTATGAATCCGAACCCTCTTC	ATGCCTCAGCAACCTCTAAC
GAPDH	ATACTGTGCACGGACAATGG	TCAGCCCATGGAATCTCTTC
